# The development of structural covariance networks during the transition from childhood to adolescence

**DOI:** 10.1038/s41598-021-88918-w

**Published:** 2021-05-04

**Authors:** Nandita Vijayakumar, Gareth Ball, Marc L. Seal, Lisa Mundy, Sarah Whittle, Tim Silk

**Affiliations:** 1 School of Psychology, Deakin University, 221 Burwood Highway, Burwood, VIC 3125 Australia; 2 Developmental Imaging, Murdoch Children’s Research Institute, Parkville, 3052 Australia; 3 Department of Paediatrics, The University of Melbourne, Melbourne, 3053 Australia; 4 Centre for Adolescent Health, Murdoch Children’s Research Institute, Parkville, 3052 Australia; 5 Melbourne Neuropsychiatry Centre, Department of Psychiatry, The University of Melbourne, Melbourne, 3053 Australia

**Keywords:** Neural circuits, Development of the nervous system

## Abstract

Structural covariance conceptualizes how morphologic properties of brain regions are related to one another (across individuals). It can provide unique information to cortical structure (e.g., thickness) about the development of functionally meaningful networks. The current study investigated how structural covariance networks develop during the transition from childhood to adolescence, a period characterized by marked structural re-organization. Participants (N = 192; scans = 366) completed MRI assessments between 8.5 and 14.5 years of age. A sliding window approach was used to create “age-bins”, and structural covariance networks (based on cortical thickness) were created for each bin. Next, generalized additive models were used to characterize trajectories of age-related changes in network properties. Results revealed nonlinear trajectories with “peaks” in mean correlation and global density that are suggestive of a period of convergence in anatomical properties across the cortex during early adolescence, prior to regional specialization. “Hub” regions in sensorimotor cortices were present by late childhood, but the extent and strength of association cortices as “hubs” increased into mid-adolescence. Moreover, these regional changes were found to be related to rates of thinning across the cortex. In the context of neurocognitive networks, the frontoparietal, default mode, and attention systems exhibited age-related increases in within-network and between-network covariance. These regional and modular developmental patterns are consistent with continued refinement of socioemotional and other complex executive functions that are supported by higher-order cognitive networks during early adolescence.

## Introduction

Converging evidence from longitudinal neuroimaging research highlights patterns of cortical thinning during childhood and adolescence^1^, reflective of synaptic pruning, myelination and/or cortical morphology^2–4^. These normative developmental trajectories do not, however, occur simultaneously across the cortex; i.e., there is regional variation in grey matter changes^5–7^. Importantly, this variability suggests that structural cortical networks are also likely to mature during this period^8^. Understanding the changing relationships between cortical regions may provide unique information beyond univariate studies of grey matter development, as covariance in structural properties is thought to arise from experience dependent plasticity and mutually trophic processes^9–11^, such that regions that fire together, wire together^12^. In particular, the transition between childhood and adolescence, and associated pubertal processes, brings about a second wave of structural “re-organization” that is second only to infancy^13–15^. However, targeted investigations of the development of structural cortical networks during this period are lacking.

Structural covariance is a multivariate analysis technique that conceptualizes how morphological properties of different brain regions relate to each other at the group-level. To do so, properties such as cortical thickness are measured for each brain region in a group of subjects, and correlations between these regional estimates are calculated for each pair of regions across the group. While most research has characterized topological properties of structural covariance across one period of the lifespan (e.g., infancy^16^, childhood/adolescence^17^, aging^18^), a smaller set of studies have shown that these properties also change over time. Studies of early childhood to late adolescence have found patterns of increasing integration (i.e., capacity to facilitate the combination of information from distributed brain regions) and decreasing segregation (i.e., capacity to facilitate specialized processing within groups of regions) of networks until late childhood, followed by inverse trajectories or plateaus during adolescence^19–21^. Those focusing on later adolescence and young adulthood have shown that the strength of overall cortical correlations decreases between 14 and 20 years of age before plateauing^8^, which is hypothesized to reflect inter-individual variability in the timing of maturation of different brain regions. Taken together, these studies provide growing evidence that the transition between childhood and adolescence may be particularly characterised by evolving structural network properties, and that these global changes may be nonlinear in pattern.

There is also emerging evidence for regional variability in covariance patterns; for example, association cortices have been found to exhibit continued increases in connectivity strength and efficiency from early childhood to young adulthood^19,21^, while paralimbic and sensorimotor cortices exhibit increases and decreases, respectively, that plateau^19^. Others have shown that neurocognitive systems (i.e., regions within functional communities) have differential developmental patterns during later adolescence and young adulthood, with the frontoparietal network exhibiting the greatest decrease in covariance^8^. Those using a lifespan approach have also found that higher-order cognitive systems exhibit structural covariance changes that differentiate young adulthood from both childhood/adolescence and older adults^22^. Prior literature has thus identified regional variability in covariance patterns when using used wide age ranges or a life-span approach. However, extending such analyses to specifically focus on the transition from childhood to adolescence may provide novel insight into prominent models of neurodevelopment that purport a mismatch between neurocognitive systems that begins during this transition period e.g. ^23,24^.

Early adolescence is also characterized by significant sex differences in biological development, which are often postulated to underlie prominent sex differences in the prevalence of psychopathology that emerge during this period^25,26^. While research has largely failed to identify such differences in structural covariance patterns in neonates^16^, young^8^ and older adults^10^, targeted investigations of the transition from childhood to adolescence may provide novel insight into sexual dimorphism. Pubertal hormone changes that support physical maturation also act on receptors in the brain^27^. Based on earlier pubertal maturation in females, some have suggested they may undergo earlier cortical^28,29^ and white matter^30^ maturation relative to males. Although a number of studies have failed to identify sex differences in cortical thinning, some support comes from research using multivariate approaches^31^. Thus, the examination of changes in network properties may provide novel insight into potentially differential patterns of cortical maturation in males and females during early adolescence.

The current study extends the literature on the network properties of brain structure during the transition from childhood to adolescence. As highlighted by Váša and colleagues^8^, prior literature has typically provided coarse-grained resolution of structural network development by categorizing participants into discrete (and wide) age groups. Arbitrary definitions of age-defined groups may also be contributing to inconsistencies in the literature. As such, we utilize a sliding window approach (as employed in recent studies^8,21^) to precisely characterize changes in structural covariance networks during this transition period (i.e., how does the relationship between structural properties of regions change over time). Our age-defined structural covariance networks are based on sliding windows of larger participant numbers and narrower age ranges relative to prior literature e.g. ^19,20^, thus increasing the robustness of our correlation estimates^32^. This was achievable with an overall sample size that was considerable for the confined developmental period that was examined. We hypothesized an increase in global connection density during the transition from late childhood and early adolescence, followed by either a plateau or decrease by mid-adolescence. We also investigated regional variation in topological properties, and characterized these changes in the context of neurocognitive networks. We hypothesized that association cortices, and particularly frontoparietal networks, may exhibit the greatest changes in covariance patterns during this period. Next, we examined associations between cortical thinning and covariance, speculating that regions that exhibit the greatest thinning during early adolescence may also have the greatest covariance with the rest of the brain. Finally, we explored sex differences in global properties and neurocognitive networks.

## Methods

### Participants

Participants were from the community in Melbourne, Australia, and were recruited into one of two longitudinal projects: i) Neuroimaging of the Children’s Attention Project (NICAP), and ii) imaging brain development in the Childhood to Adolescence Transition Study (iCATS). NICAP participants were recruited as typically developing controls into a study of a community-based cohort of children with and without ADHD. Further details on the NICAP and iCATS samples is presented in Silk et al.^33^ and Simmons et al.^34^, respectively. Exclusion criteria for these analyses included MRI contraindications, developmental disability, history of a neurological or serious medical disorder (e.g., diabetes, kidney disease), and psychotropic medications. For both cohorts, written informed consent was obtained from the parent/guardian of all participants. Ethics approval was granted by the Royal Children’s Hospital Human Research Ethics Committee, Melbourne (NICAP #34,071; iCATS #32,171). The iCATS protocol was additionally ratified by the University of Melbourne Human Research Ethics Office (#1238745), and the NICAP protocol was ratified by the Deakin University Human Research Ethics Office (#2016–394). Methods were performed in accordance with these approved protocols.

The NICAP sample underwent up to 3 repeated assessments between the ages of 9.5 and 14.5 years, with approximately 18-month intervals (M = 1.432, SD = 0.222, 1.021–2.330 years) between assessments. The iCATS sample underwent 2 repeated assessments between the ages of 8.5 and 13.5 years, with approximately 36-month intervals (M = 2.763, SD = 0.243, 2.158–3.344 years) between assessments. The two samples did not differ in sex (χ^2^ = 1.342, df = 1, *p* = 0.247), pubertal stage (based on parent-report Sexual Maturity Scale^35^; Mean: iCATS = 1.282, NICAP = 1.316, t_(161)_ =  − 0.431, *p* = 0.666), or intelligence (based on Wechsler Abbreviated Scale of Intelligence—Matrix Reasoning T-score; Mean: iCATS = 54.096, NICAP = 52.607, t_(179)_ = 1.225, *p* = 0.222). However, the iCATS sample was significantly younger than the NICAP sample at baseline (Mean: iCATS = 9.556, NICAP = 10.425; t_(157)_ =  − 14.928, *p* < 0.001) and had higher socioeconomic status (based on Socio-Economic Indexes for Areas—Index of Relative Socio-economic Advantage and Disadvantage, based on Australian census data; Mean: iCATS = 1056.175, NICAP = 1018.326; t_(198)_ = 4.887, *p* < 0.001).

Following exclusions during quality-control (see below for further detail), the final sample comprised 366 scans from 192 participants (96 females, 90 NICAP) aged 8.5–14.5 years, which was used to create cross-sectional windows (see “Statistical analyses” for further detail). Specifically, 59 participants (28 males) provided a single observation, 92 (46 males) provided two observations, and 41 (22 males) provided three observations. For further breakdown of these numbers by cohort, and distributions of age and sex at each wave, refer to Vijayakumar et al.^36^).

### MRI acquisition and processing

Neuroimaging data for both projects were acquired on a 3 T Siemens scanner (Siemens, Erlangen, Germany) at the Murdoch Children’s Research Institute in Melbourne, Australia. Participants completed a mock-scan prior to their actual scan at wave 1 (and was repeated at subsequent waves if the participant wished or researcher deemed it appropriate). They were also given information on MRI (including a video) prior to participating in order to familiarize them with the procedure and minimize anxiety as much as possible. Both waves of iCATS, and waves 1 and 2 of NICAP, were collected on a TIM Trio scanner. The final wave of NICAP was collected after an upgrade to a MAGNETOM Prisma scanner, which has been accounted for in statistical modelling. Refer to the Vijayakumar et al.^36^ for further detail of an investigation into potential cortical differences related to scanner upgrade, which found that only one region (the right insula) exhibited significant differences when comparing pre- and post-upgrade estimates in a sample of age- and sex-matched participants (N = 22).

Participants lay supine in a 32-channel head coil during the MRI scan. Structural T1-weighted images were acquired as follows: *iCATS*. MPRAGE with repetition time = 1900 ms, echo time = 2.24 ms, flip angle = 9**°,** field of view = 230 mm^2^, resulting in 176 contiguous slices with voxel dimensions 0.9 mm^3^. *NICAP*. MEMPRAGE with repetition time = 2530 ms, echo time = 1.77, 3.51, 5.32, 7.2 ms, flip angle = 7**°,** field of view = 230 mm^2^, resulting in 176 contiguous slices with voxel dimensions 0.9 mm^3^.

T1-weighted images were processed through FreeSurfer 6.0, a freely available image analysis suite for cortical reconstruction and volumetric segmentation (http://surfer.nmr.mgh.harvard.edu/). Specifically, images were processed with the submillimeter reconstruction^37^ and the longitudinal stream that creates an unbiased within-subject template space from all available data using robust, inverse consistent registration. The template is used as an estimate to initialize segmentation processes for each time point, providing common information regarding anatomical structures, and has been found to significantly increase reliability and statistical power^38,39^. The quality of i) raw images and ii) (longitudinal) cortical reconstructions was visually inspected and rated for all scans. Raw images were rated on a 4-point scale for “ringing” (1: no ringing; 2: slight ringing restricted to a small cortical area; 3: more ringing extending into white matter and/or covering more brain regions; 4: extensive ringing) and “blurriness” (1: sharply defined images; 2: slight blurriness; 3: or considerable blurriness; 4: blurring throughout). Ratings of “3” and “4” on either scale were excluded. Processed images were rated on a 3-point scale on the accuracy of the white and pial surfaces (1: near perfect reconstruction; 2: minor reconstruction issues limited to small areas of the brain; 3: poor reconstruction with consistent under-estimation of white matter or extensive areas of CSF included as grey matter). Ratings of “3” were excluded. Images were also processed through MRIQC (v0.14.2) to supplement the visual inspection^40^. This resulted in the exclusion of a total of 37 scans from 34 participants (i.e., 3 participants had 2 scans removed). No manual edits were made to the remaining (included) data (further details of the quality control procedure have been reported previously^36^) . Mean cortical thickness estimates from 360 regions of the Human Connectome Pipeline’s multimodal parcellation atlas (HCP-MMP1)^41^ were extracted^42^ and used in subsequent analyses. Supplemental analyses examined mean cortical thickness of 62 regions of the Desikan Killiany Tourville (DKT) atlas^43^.

### Statistical analyses

#### Structural covariance networks (SCN)

**Figure 1:**
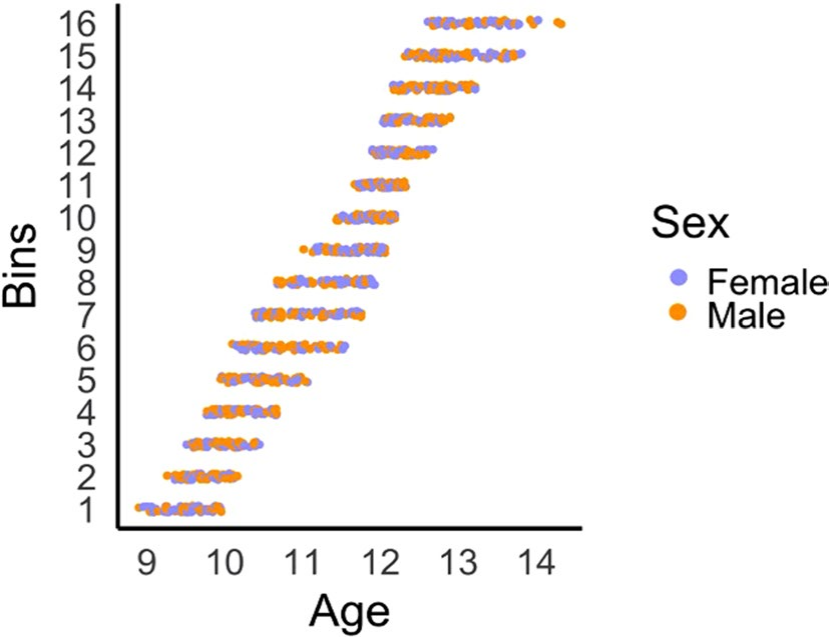
Illustration of 16 age-bins for the sliding window configuration with a bin size of 80 and step of 25%. The number of age-bins varied between 12 and 23 across the 9 sliding window configurations. Structural covariance networks (adjusted for sex, cohort, scanner) were created for each age-bin, and the median age was assigned to each network. Individual scans contributed to multiple age bins, but only one scan per participant was included in each bin.

The sliding window approach involves creating a series of overlapping “bins” of participants while incrementally sliding across the age range of this sample. Bins were defined by *i)* an equal sample size, and *ii)* an incremental step size. As this sliding window approach requires an arbitrary definition of sample and step sizes, we ran analyses across a series of these parameters. Bin sizes of n = 70, 80 and 90, in conjunction with overlap of 70%, 75%, and 80% of the sample (i.e., incremental age-based “steps” of 20%, 25%, and 30%), were examined. Every combination of bin and step sizes were used, thus producing 9 configurations. A visual representation of the data for one sliding window configuration is presented in Fig. 1.

For each of these 9 sliding window configurations, structural covariance networks were created for each age-bin, and the median age of each age-bin was assigned to the respective matrix. Each participant contributed a single scan to each age-defined network. In instances where more than one scan from a participant fell into a given age bin, the scan closest to the median age was selected. The 3 bin sizes and 3 step parameters were chosen based on the trade-off between bin size and repeated assessments per participant within each bin. In other words, larger bin sizes had wider age ranges, and thus participants were more likely to contribute two scans to each age-bin.

Given sample differences in demographics and scanner protocols described above, prior to creating structural covariance networks for each bin, we performed linear regression, modelling cortical thickness of each region as a function of sex, cohort (iCATS, NICAP) and scanner (pre-upgrade, post-upgrade) across the full sample. The standardized residuals of this model were calculated using the “rstandard” function in R, and used to create the structural covariance networks. That is, for the subsample of participants in each bin, we correlated cortical thickness across 360 regions defined by the HCP-MMP1 parcellation, and as supplemental analyses for the 62 regions defined by the DKT parcellation.

There are concerns of noise when estimating structural covariance networks from small sample sizes, although knowledge of appropriate sample size remains limited^32^. Prior research has used a probabilistic bootstrap thresholding procedure to address this issue^8^, whereby connections are retained on the basis of a criteria of connection “likelihood” rather than connection weights or fixed edge density. The bootstrap thresholding approach identifies the most statistically robust connections in a network, which are least likely to represent false positives. Within each age-bin, 1000 sets of participants were sampled with replacement, to re-estimate the structural network. We retained edges that were consistently positive across bootstraps (at false discovery rate [FDR] corrected *α* = 0.05) and set the remaining edges to zero (note, negative edges were identified in an average of 0.04% (range: 0.06–0.1) of the edges across all window configurations). Thresholded networks were binarized prior to graph analyses.

#### Graph metrics

Graph metrics of each thresholded and binarized SCN were examined using global, nodal and modular properties. We focused on basic metrics as bootstrap thresholding produces variable density for each network, which can confound differences between networks when comparing higher-order graph metrics^44^. Thus, we examined global density (i.e., percentage of total connections) and node degree (i.e., number of connections per region). Hubs were defined as nodes with standardized degree greater than 1 (i.e., > 1SD from the mean of a given SCN)^45,46^. SCNs were also decomposed into a functional modular structure using the Yeo 7 parcellation scheme^47^, with each cortical region of the HCP-MMP1 atlas assigned to one Yeo module based on maximum overlap of vertices^48^. For this structure, we calculated *i)* intra-modular density, defined as the density of connections within each module (i.e., number of connections between nodes within a module, relative to all possible connections) and *ii)* inter-modular density, defined as the density of connections between each pair of modules (i.e., number of connections between nodes in each pair of modules, relative to all possible connections)^8^. Given changes in global network properties over age windows, these modular metrics were normalized (i.e., divided by) global density of the respective SCN.

#### Development of structural covariance networks

Changes in the topological properties of SCNs between 8 and 15 years of age were examined with generalized additive models (GAM), using the “mgcv” package^49^ in R^50^. Specifically, a given property of each bin was assigned to the median age of participants within the bin. We then examined changes in the property with respect to the (median) age. In a “smooth” model, (e.g.) density was predicted by a smooth age term with a basis function of 3 (i.e., the maximum possible degrees of freedom allowed for the smooth term) given the constrained time frame (e.g., density ~ s(age, k = 3)). This model was compared to a “linear” model (e.g., density ~ age) and a “null” model (e.g., density ~ 1). All models were examined with maximum likelihood (ML) estimation, and model comparisons were used to identify best-fitting developmental trajectories. Model comparisons were based on AIC values, with more complex models selected if AIC was at least 3 less than all lower-order models (i.e., a “smooth” model was only selected if AIC was lower than both “linear” and “null” models).

This model fitting procedure was first used to examine changes in the mean correlation of unthresholded networks and global density of thresholded networks. Second, we examined changes in the connectivity of “hubs” by modelling age-related changes in the (standardized) degree of hub regions. We limited these analyses to regions that were classified as hubs in at least two age-bins, in order to minimize potential noise from regions classified as hubs in one age-bin alone. Third, we examined changes in (normalized) intramodular and intermodular density of functional networks using the same model fitting procedure. We additionally checked the significance of smooth age terms when correcting for multiple comparisons using Benjamini & Hochberg FDR correction^51^ in nodal and modular analyses.

**Figure 2:**
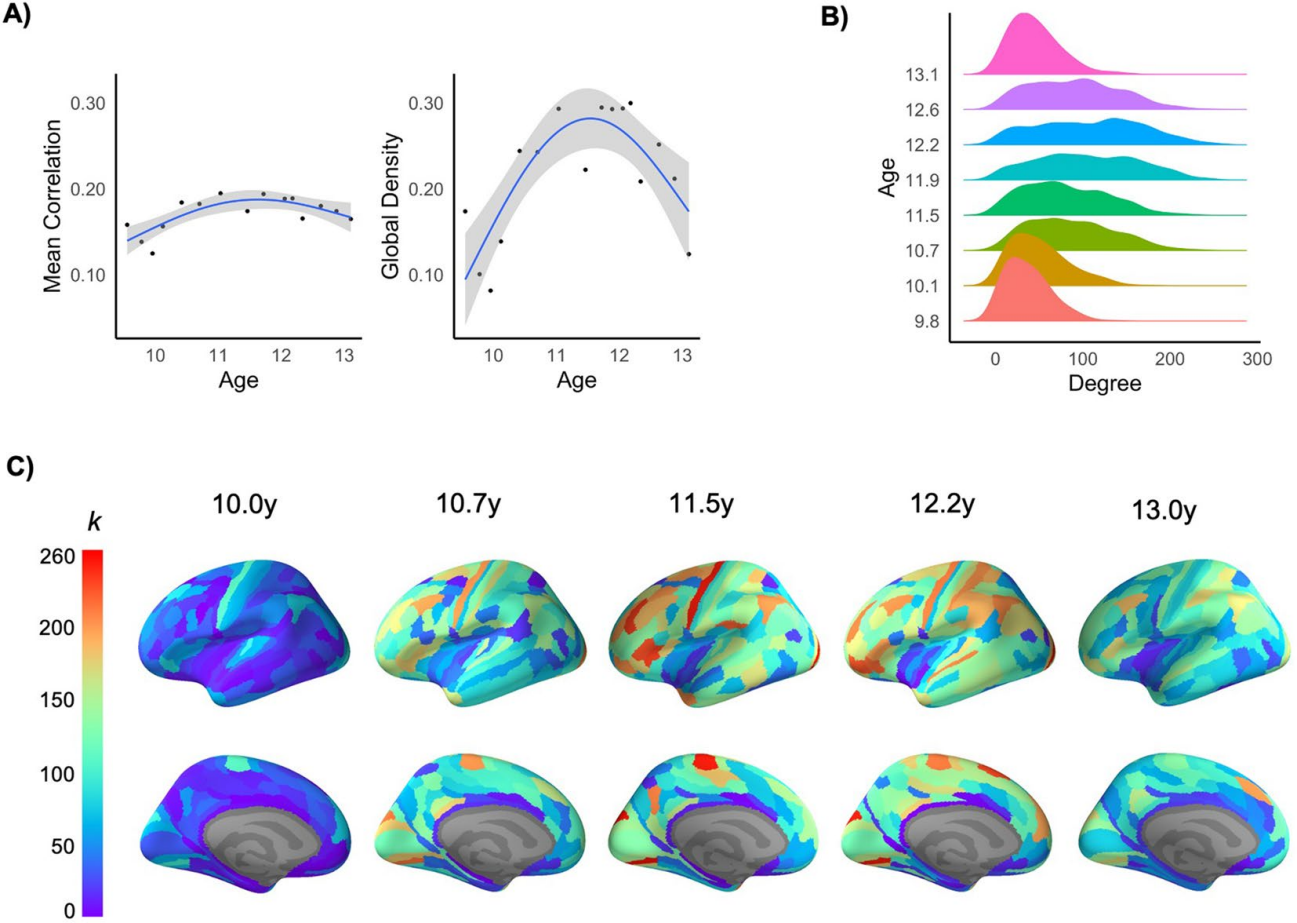
(**A**) Changes in global properties of SCNs across age-bins, illustrating nonlinear change in mean strength of positive correlations across the network, and global edge density of the bootstrap-thresholded network (Refer to Figures S2 and S3 for illustration of changes in each sliding window configuration). (**B**) Changes in degree distribution of bootstrap-thresholded networks, and (**C**) Nodal degree (*k*) across the left cortex at 5 age-bins between late childhood and mid-adolescence. Effect sizes are illustrated for the sliding window size of 80 and step of 25%.

**Figure 3:**
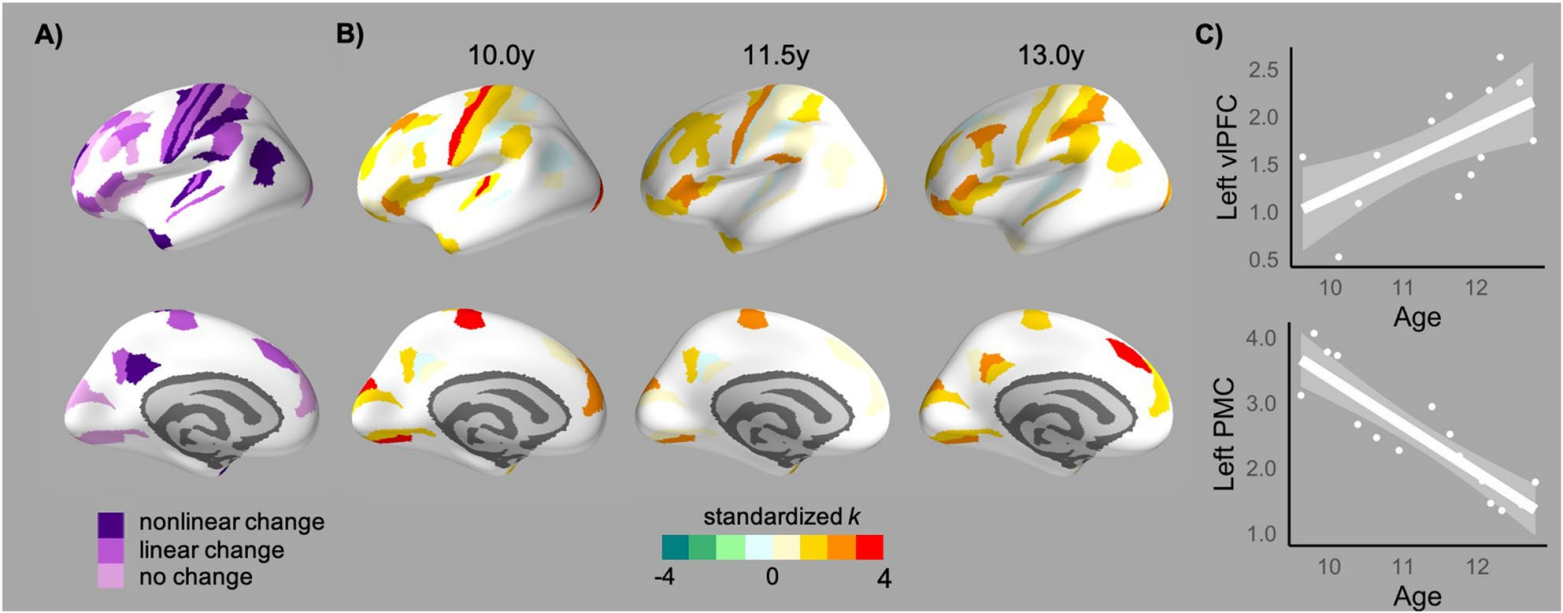
(**A**) Age-related change in standardized degree of hubs regions (as identified in more than 50% of sliding window configurations; refer to Supplementary Fig. S4 for illustration of changes in each window configuration). (**B**) Patterns/directions of change illustrated by standardized degree of these hubs in early, middle and late age-bins. (**C**) Prototypic changes in standardized degree (k) within the left ventrolateral PFC (vlPFC) and left primary motor cortex (PMC). Effect sizes in (**B**) and (**C**) are illustrated for the sliding window size of 80 and step of 25%.

GAM models were run for 9 combinations of bin and step size, and only findings that were identified in more than 50% of these sliding window configurations are presented. Model coefficients and illustrations of significant developmental patterns are presented for the bin size of 80 and 25% step, as it represents the median of all 9 configurations that were examined. Whole brain maps in Figs. 2C and 3 (as well as Supplementary Figures S1C and S4) were created using PySurfer v0.10.0 (https://pysurfer.github.io/).

#### Associations with cortical thinning

Rates of thinning were calculated for each region using linear mixed models using the “lmer” package in R. We modelled linear trajectories based on prior work examining cortical development in this dataset^36^: Y = Intercept + d_i_ + β_1_ (sex) + β_2_ (cohort) + β_3_ (scanner) + β_4_ (age) + e_i_. Models were conducted within each i^th^ subject, with a random intercept (d_i_) to account for the repeated observations per subject. The e_i_ represents the normally distributed residual error term. The β coefficients for age were extracted as effect sizes of regional thinning. In order to understand whether rates of cortical thinning were associated with covariance properties in mid-adolescence, we correlated the β coefficients with standardized node degree in the oldest age-bins. Significant associations were followed up by correlating β coefficients to node degree of the youngest age-bins, to determine whether potential associations were unique to mid-adolescence (and thus reflective of changes with age). To calculate significance of these correlations, BrainSMASH was used to simulate 1000 surrogate brain maps that preserve the spatial autocorrelation of the original cortical map (based on the geodesic distance matrix of parcels in each hemisphere)^52^. Correlations between thinning and covariance in each hemisphere were then calculated for each surrogate map, and non-parametric *p* values were calculated as the proportion of surrogate maps that generated correlations equal to or greater than the empirical value.

#### Exploratory sex differences

Using the same sliding window configurations as above, SCNs were recreated for males and females within each age-window. To do so, linear regression was first run within each sex to remove the influence of variables of non-interest (cohort (iCATS, NICAP) and scanner (pre-upgrade, post-upgrade)) from cortical thickness. Next, SCNs for males and females were created for each age-window. They were bootstrap-thresholded, binarized, and graph metrics were calculated. Generalized additive models examined sex differences in age-related trajectories for mean correlations and global density of SCNs. Finally, we examined changes in (normalized) intramodular and intermodular density of functional networks within each sex, and GAMs examined “null”, “linear” and “smooth” change. Sex differences in modular density were not statistically examined as normalized metrics are dependent on global density, which differed between males and females.

## Results

### Global development

Generalized additive modelling of non-thresholded SCNs revealed nonlinear change in edge strength (i.e., mean of all correlations) between the youngest and oldest age windows, characterized by a “peak” around 11.5 years of age. This pattern of nonlinear change in mean correlation was consistent across 8 out of 9 sliding window configurations. When focusing on the most statistically robust connections (i.e., bootstrap-thresholded and binarized SCNs), GAMs revealed a similar pattern of nonlinear change with maximum global edge density at 11.5 years of age (Fig. 2A). This pattern of nonlinear change was consistent across all 9 configurations. Thresholded SCNs also exhibited increased variability in degree (i.e., degree distribution) at the middle age windows (~ 11.5 years) relative to the youngest and oldest windows (Fig. 2B). Refer to Supplementary Table S1 for model fit and coefficients. Nonlinear changes in global metrics over the age windows was replicated for the DKT parcellation, although degree distribution was negatively skewed (i.e., most of the 62 regions were correlated with one another; see Supplementary Fig. S1).

### Nodal development

At a regional level, a similar pattern of nonlinear change was identified for nodal degree (Fig. 2C); across the cortex, regions exhibited a pattern of increasing numbers of connections between the youngest and middle age windows (i.e., roughly 9.5 to 11.5 years of age), followed by reductions through to the oldest age-window (~ 13.5 years). Hubs, defined as regions with high degree (> 1 SD) in at least two age windows, were identified across sensorimotor and association cortices during early adolescence (Fig. 3). While GAMs revealed that a number of these hubs did not exhibit change in standardized degree across the age-bins, significant decreases were identified in motor regions and increases were identified in multiple frontal and parietal regions (see Supplementary Table S2 for model comparisons and coefficients). Additionally, correlational analyses conducted across the entire parcellation revealed that rates of cortical thinning were associated with standardized degree in the oldest age-bin, such that regions exhibiting greater thinning had greater degree (Left hemisphere: *r* =  − 0.270, *p* = 0.011, 9/9 window configurations; Right hemisphere: *r* =  − 0.241, *p* = 0.044, 6/9 configurations). However, there were no such associations in the youngest age-bin (Left: *r* =  − 0.160, *p* = 0.182, 9/9 configurations; Right: *r* =  − 0.185, *p* = 0.171, 9/9 configurations).

### Modular development

**Figure 4:**
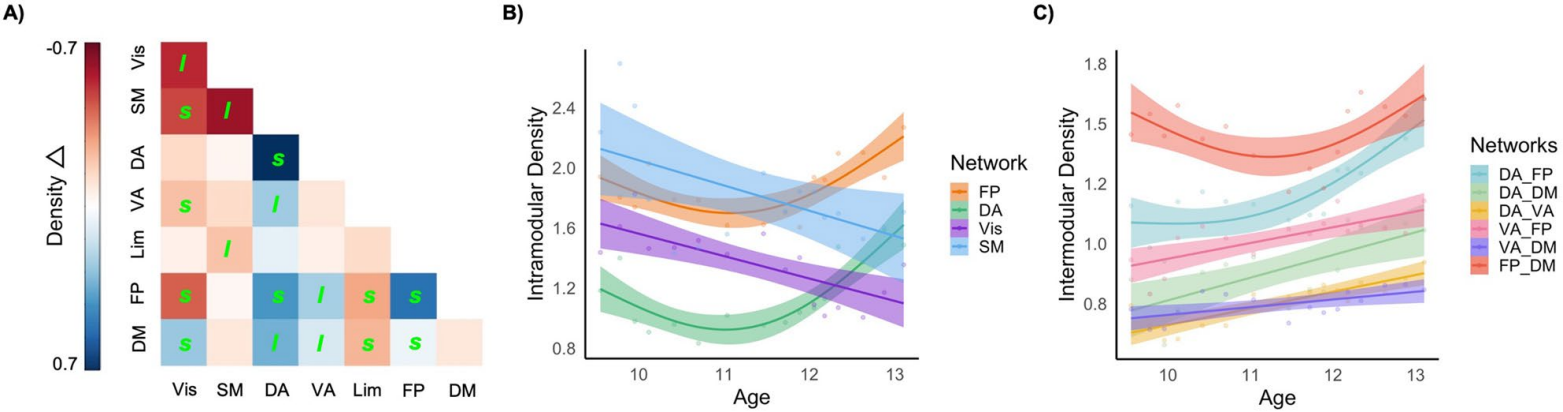
Changes in the density of connections for functional networks. (**A**) Change in “normalized” density of connections between and within networks (*l* and *s* indicate significant linear and nonlinear (smooth) trajectories, respectively). Effect size is the difference between maximum and minimum density for the sliding window size of 80 and step of 25%. Significant developmental trajectories of certain networks for normalized (**B**) intramodular density and (**C**) intermodular density, also illustrated for the sliding window size of 80 and step of 25%. *DA* dorsal attention, *DM* default model, *FP* frontoparietal, *Lim* limbic, *VA* ventral attention, *Vis* visual.

In the context of functional communities, “peaks” were identified for mean density across all modules, consistent with patterns of global density. To further understand community-level changes, we examined “normalized” metrics of modular density that accounted for global density. Normalized intramodular density (i.e., connections of regions within a network) of the dorsal attention and frontoparietal networks exhibited nonlinear increases over age-windows (Fig. 4A,B), while that of the visual and somatomotor networks exhibited linear decreases over age. Normalized intermodular density (i.e., connections of regions between networks) exhibited predominantly linear increases between the ventral attention, dorsal attention, frontoparietal, and default mode networks (see Fig. 4A,C). Comparatively, changes involving the visual network were largely characterized by “peaks”, while those involving the limbic network were characterized by “troughs”. Model comparisons and coefficients for modular density are presented in Supplementary Table S3. Post-hoc illustration of differences between hemispheres are presented in Supplementary Fig. S5.

### Sex differences

**Figure 5:**
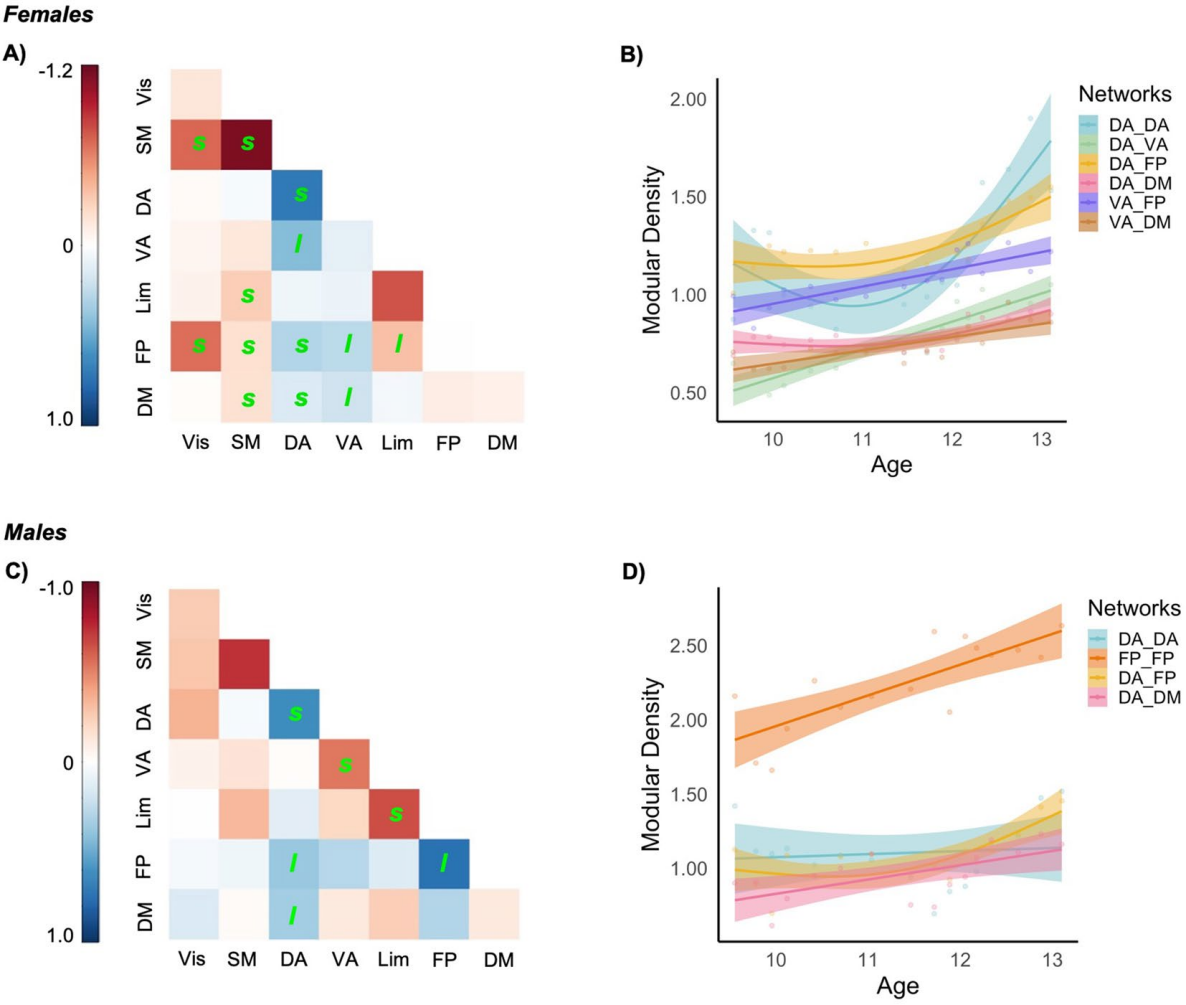
Change in “normalized” density of connections between and within networks in females (**A**) and males (**C**); *l* and *s* indicate significant linear and nonlinear (smooth) trajectories, respectively. Effect size is the difference between maximum and minimum density relative to minimum density for the sliding window size of 80 and step of 25%. Significant developmental trajectories of certain networks illustrated in females (**B**) and males (**D**), also illustrated for the sliding window size of 80 and step of 25%. *DA* dorsal attention, *DM* default model, *FP* frontoparietal, *Lim* limbic, *VA* ventral attention, *Vis* visual.

Exploratory analyses failed to identify any consistent sex differences in age-related changes of non-thresholded mean correlation (“smooth” AIC: − 143, “smooth * sex” AIC: − 145) and thresholded global density (“smooth” AIC: − 136, “smooth * sex” AIC: − 137). Next, changes in the density of connections between functional communities were examined within each sex, normalized by their respective global densities. In females, increases in intra- and inter-modular density were limited to the dorsal and ventral attention networks, particularly in connection with the frontoparietal and default mode networks. In comparison, a number of nonlinear reductions were identified for the visual and somatomotor networks (Fig. 5A,C). In males, increases in modular density were present for the dorsal attention and frontoparietal networks, while decreases were limited to intramodular connections of ventral attention and limbic networks (Fig. 5B,D). See Supplementary Table S4 for results.

## Discussion

The current investigation revealed age-related changes in structural covariance networks during the transition from childhood to adolescence. As hypothesized, there was a “peak” in global covariance between 9.5 and 14.5 years of age based on correlations in regional thickness across subjects. There was also regional variability beyond this global pattern, with association cortices exhibiting greater increases in covariance. Relatedly, regions within higher-order neurocognitive systems exhibited greater within-network and between-network covariance with age, compared to sensorimotor networks. Exploratory analyses also indicated that these patterns were more prominent in females relative to males. Finally, as hypothesized, regions exhibiting the greatest thinning during this period had the greatest covariance with the rest of the brain.

Findings indicate that the transition from childhood to adolescence is characterized by global increases in structural covariance, followed by reductions into mid-adolescence. This pattern was identified for the strength of non-thresholded connections (i.e., mean correlations), as well as the density of thresholded connections. Similar nonlinear trajectories have been identified during this period for mean correlations^19,21,53^ and mean local efficiency (a measure of communication between the nodes)^19^. These global “peaks” may be reflective of a transient period of convergence in anatomical properties across the cortex during early adolescence, followed by a divergence that reflects greater inter-individual variability in the rates or timing of regional maturation over the course of the second decade. Somewhat consistent with this speculation, others have shown that global covariance continues to decrease through late adolescence before plateauing in the early 20 s, which corresponds to the protracted maturation of association cortices^8^. Future research is needed to understand whether such a divergence may be related to the onset of socioemotional problems during adolescence, as purported by mismatch models of neurodevelopment^24,54^.

Our analyses also highlighted regional differences in covariance properties of sensorimotor and association cortices during early adolescence. Examination of (standardized) degree revealed the prominence of sensorimotor regions as highly connected “hubs” by late childhood. The connectivity of visual hubs remained consistent across early adolescence, while motor hubs exhibited significant reductions with age. Although there were some hub regions present in associations cortices by late childhood, the extent and strength of hubs within frontal and parietal cortices increased with age. These results are consistent with prior literature that has highlighted the prominence of hubs within association cortices during adolescence^19^, although this has been limited to parietal regions in some studies^20^. Findings are also supported by regional variability in group-level trajectories, with frontal, parietal and temporal cortices exhibiting greater changes than occipital and motor cortices in adolescents^55,56^ and exhibiting later maturation in macaques^57^. Moreover, increasing covariance was mostly limited to regions that support abstract higher-order cognitive skills that continue to mature during adolescence^58^. Interestingly, others have shown that mean correlation strength is related to (group-level) working memory during adolescence^21^. However, our findings suggest potential value in examining the contribution of specific regions’ covariance properties in relation to cognitive maturation during early adolescence.

When accounting for the global “peaks” in density, there were also age-related (linear) increases in intramodular (within network) correlations in frontoparietal and ventral attention networks, suggesting that regions within these networks become increasingly connected with one another relative to average connectivity of the cortex. There were also age-related linear increases in intermodular (between network) connections between the ventral attention, dorsal attention, frontoparietal and default mode networks. Comparatively, there was a decoupling of connections with visual and somatomotor networks with age. Taken together, higher-order cognitive networks appear to undergo coordinated structural development that supports their specialization and segregation across adolescence, within the context of a generalized divergence across the cortex. These findings are consistent with seed-based analyses of covariance that have found primary sensory and motor networks to be well-developed in early childhood, and later maturation of salience and executive control networks during adolescence^17^. Others have found that higher-order networks continue to mature into young adulthood, with the frontoparietal network exhibiting the greatest reduction in covariance during the late teens and early 20 s, whilst the default mode and ventral attention networks are the last to reach adult levels of maturity^8^. A similar developmental pattern is also postulated for functional connectivity, with visual and sensorimotor areas developing earlier^59^ and exhibiting less variability in their trajectories^60^ than other networks. Findings are also consistent with continued maturation of between-network connectivity of task-positive (frontoparietal, attention) and task-negative networks (default mode) during adolescence^61–64^. Developmental patterns in both nodal and modular properties are therefore consistent with earlier mastery of basic sensory and motor skills, but continued refinement of emotion regulation, social cognition and other complex executive functions that are supported by frontoparietal, default mode, and attention networks.

The exact mechanisms that underlie structural covariance networks remain uncertain, but it is frequently postulated to arise from coordinated functioning of distributed brain regions. In support, there is some overlap between structural covariance and functional networks^65,66^. Work in neonates has also found that SCNs develop later than functional networks, suggesting that coactivation of functional networks may guide the development of SCNs^16^. There is also partial convergence with structural connectivity networks^67^, suggesting a role of mutually trophic effects mediated by underlying axonal connections^10^. Others have highlighted similarities with maturational covariance networks (i.e., correlations of longitudinal regional trajectories)^65^, as well as coordinated gene expression during brain development^68,69^. Moreover, higher covariance between regions at shorter distances may also relate to shared gene expression from common embryonic origins^69^. Importantly these mechanisms are not mutually exclusive, as trophic, genetic and neurodevelopmental processes are most likely interconnected influences on SCNs^9,11^.

Less is known of the mechanisms that may contribute to *developmental changes* in structural covariance. However, we found that regions exhibiting greater cortical thinning had greater degree by mid-adolescence. As rates of cortical thinning were not associated with node degree during late childhood, findings imply that thinning specifically contributes to changes in covariance networks between late childhood and mid-adolescence. During later adolescence, greater cortical thinning has conversely been show to relate to more reductions in nodal degree^8^. As association cortices exhibited the greatest cortical thinning across both datasets, it appears that underlying neural mechanisms, such as synaptic pruning and myelination, may have nonlinear effects on the network properties of these regions across the extended period of adolescence. Váša and colleagues^8^ also show that intracortical myelination has stronger associations with regional changes in covariance than cortical thinning, highlighting the need for multimodal analyses to unpack the evolving relationship between structural connectivity and structural covariance networks during development.

Exploratory investigations of sexual dimorphism failed to identify consistent differences in age-related changes of global covariance (i.e., mean correlation and global density), although nonlinear age-related differences in a number of sliding window configurations suggest that females may have steeper “peaks” than males in global density during early adolescence. Given these inconsistencies, continued research with larger sample sizes per group, within this targeted age range, is thus needed to further investigate sex differences. Our finding of overall greater mean correlation in females is also inconsistent with prior research that has found greater correlations of subcortical volumes in males^70^, suggesting that sex differences in anatomical networks may differ by regions-of-interest and/or morphologic properties during development. When accounting for potential global differences, females exhibited a general pattern of increased covariance of (dorsal and ventral) attention networks with one another and the frontoparietal and default mode networks, while males had fewer such increases that were concentrated in the dorsal attention and frontoparietal network. Moreover, females exhibited a number of decreases in the covariance of primary sensorimotor networks. Prior seed-based analysis has shown sex-specific associations between testosterone levels and prefrontal-hippocampal covariance during adolescence^71^. Moreover, covariance has been found to differ between peri- and post-menopausal women^72^, and is also related to estradiol levels in adults^73^. Together with the lack of sex differences in the structural covariance of neonates and later adolescents/young adults^8,16^, it appears that sex differences in covariance may be specific to periods of rapidly changing hormone levels. Our pattern of findings may also be suggestive of earlier maturation in females, as they had more extensive segregation of later maturing higher-order cognitive networks, and also exhibit “decoupling” of earlier maturing sensorimotor networks. However, it is important to note that differences in modular density are qualitative, and further investigations incorporating hormones and a more extended period of adolescence is needed to test our hypotheses.

### Limitations

Our findings need to be considered in light of certain strengths and limitations. The current investigation of age-related changes infers developmental processes from group-level networks. As discussed above, changes in cross-sectional correlations are suggestive of individual differences in developmental trajectories and longitudinal research is needed to fully understand these underlying neurodevelopmental processes. Our analyses are also dependent on the configuration of age-windows based on window and step/overlap sizes. However, we conducted statistical analyses on a range of window and step sizes so as to identify results that were consistent across the majority of configurations. Windows were also comprised of large participant numbers and narrow age ranges relative to prior literature, but further increases in sample size will continue to decrease noise within age-defined SCNs, and also create additional age-bins/estimates to model age-related changes. Another limitation of the sliding window approach is that each window’s topological properties that are modelled within GAMs are not independent of one another. The MRI scanner was also upgraded between waves in one of the cohorts, but we additionally modelled this upgrade as a covariate in our analyses. Moreover, comparison on age-matched participants pre- and post- scanner upgrade failed to identify significant differences^36^. We also note that stringent quality control procedures were undertaken to minimize the influence of head motion, but future studies with estimates of head motion for T1-weighted images are needed to confirm findings while statistically controlling for motion confounds. Finally, our sex differences are considered exploratory given the smaller sample size when differentiating males and females, although we note that similar sample sizes have been used frequently in the literature. Nevertheless, given concerns of the reliability of estimates for small samples^32^, future work with larger samples of this age range are needed to corroborate our findings. The incorporation of pubertal measures may also provide novel insight into the biological processes that may underlie the identified changes in structural covariance networks during this period.

## Conclusions

The transition from childhood to adolescence is characterized by nonlinear trajectories with “peaks” in global covariance of cortical thickness. While occipital and motor regions are more highly connected hubs during late childhood, the prominence of frontal and parietal regions as hubs increases with age. Beyond global patterns, attention, frontoparietal and default mode networks exhibit increasing covariance, suggesting greater specialization and segregation of regions that support higher-order cognitive processes during early adolescence.

## Supplementary Information

Supplementary Information

## Data Availability

The datasets analysed in the current study are not publicly available as we do not have consent from participants in the Imaging of the Children’s Attention Project (iCATS) cohort to share individual data. However, it can be made available from the corresponding author on reasonable request. Custom analysis scripts can be found at https://github.com/nandivij/struct_cov_dev.
