# Supplementary material for: The development of structural covariance networks during the transition from childhood to adolescence
Source: Sci Rep. 2021 May 4;11:9451. doi: 10.1038/s41598-021-88918-w (PMC8097025; doi:10.1038/s41598-021-88918-w)
Supplement: Supplementary file 1 — Supplementary Information [file 41598_2021_88918_MOESM1_ESM.pdf]

The development of structural covariance networks during the transition from childhood to adolescence

Nandita Vijayakumar, Gareth Ball, Marc L Seal, Lisa Mundy, Sarah Whittle, Tim Silk

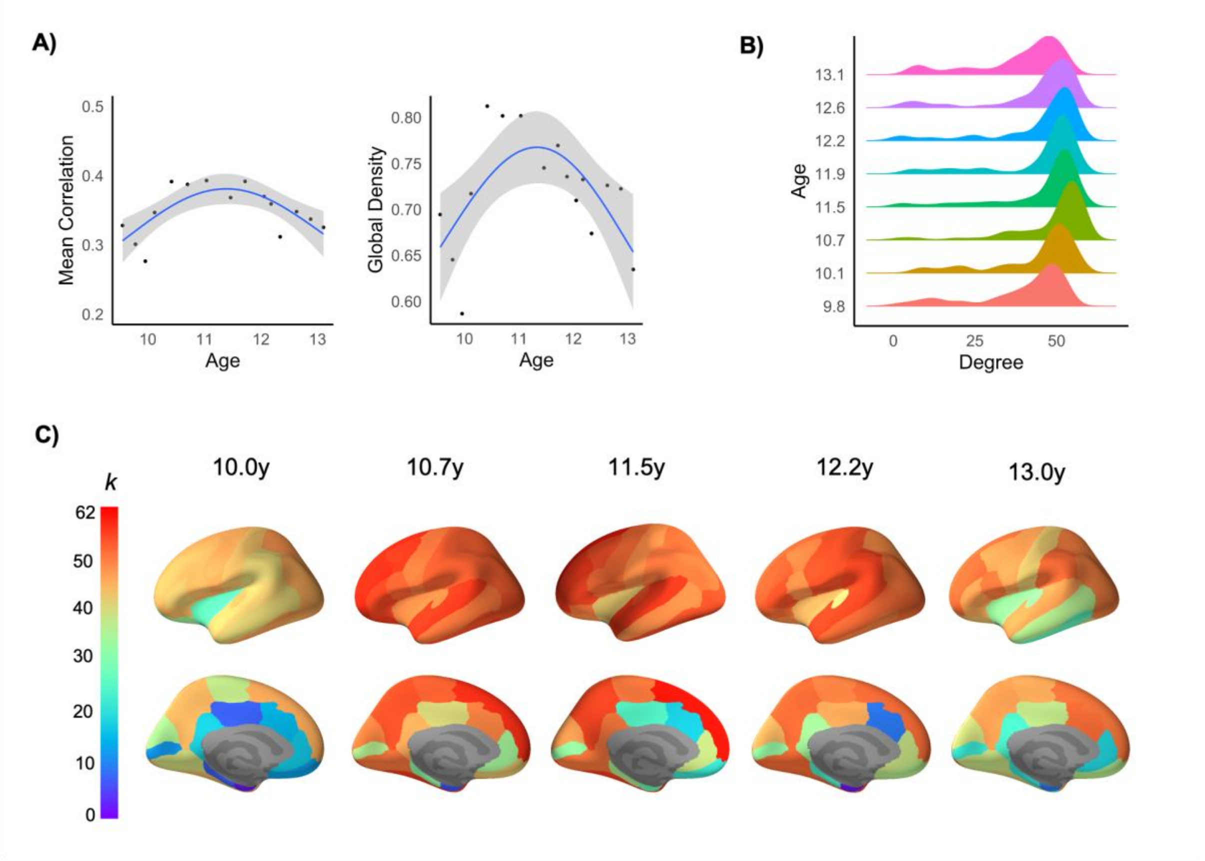

Figure S1. A) Changes in global properties of SCNs for the DKT parcellation, illustrating nonlinear change in mean strength of positive correlations across the network, and global edge density of the bootstrap-thresholded network. *B)* Changes in degree distribution of bootstrap-thresholded networks, and *C)* Nodal degree (*k*) across the left cortex at 5 age-bins between late childhood and mid-adolescence. Effect sizes are illustrated for the sliding window size of 80 and step of 25%.

Table S1. Model comparisons and coefficients for global metrics

	Across configurations		Bin 80, Step 25%						
			AIC			s(Age)			
	Best Fit	#	Null	Linear	Smooth	EDF	Ref DF	F	p
HCP-MMP1									
Mean correlation	Smooth	8	-71.36	-73.46	**-81.63**	1.927	1.995	9.650	0.003
Global density	Smooth	9	-35.05	-36.13	**-49.07**	1.957	1.998	13.521	0.001
DKT									
Mean correlation	Smooth	8	-54.30	-52.59	**-61.90**	1.937	1.996	6.730	0.011
Global density	Smooth	7	-40.24	-38.25	**-45.72**	1.918	1.993	4.893	0.026

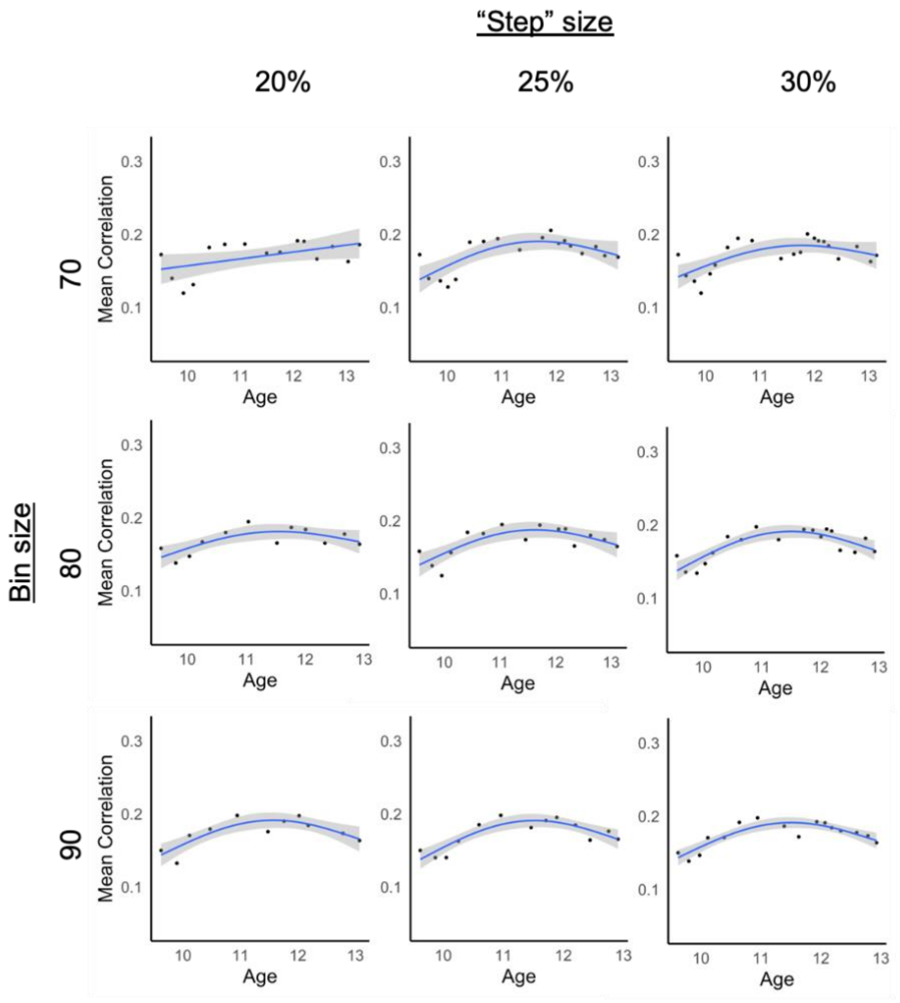

Figure S2. Age-related changes in mean correlation for each sliding window configuration.

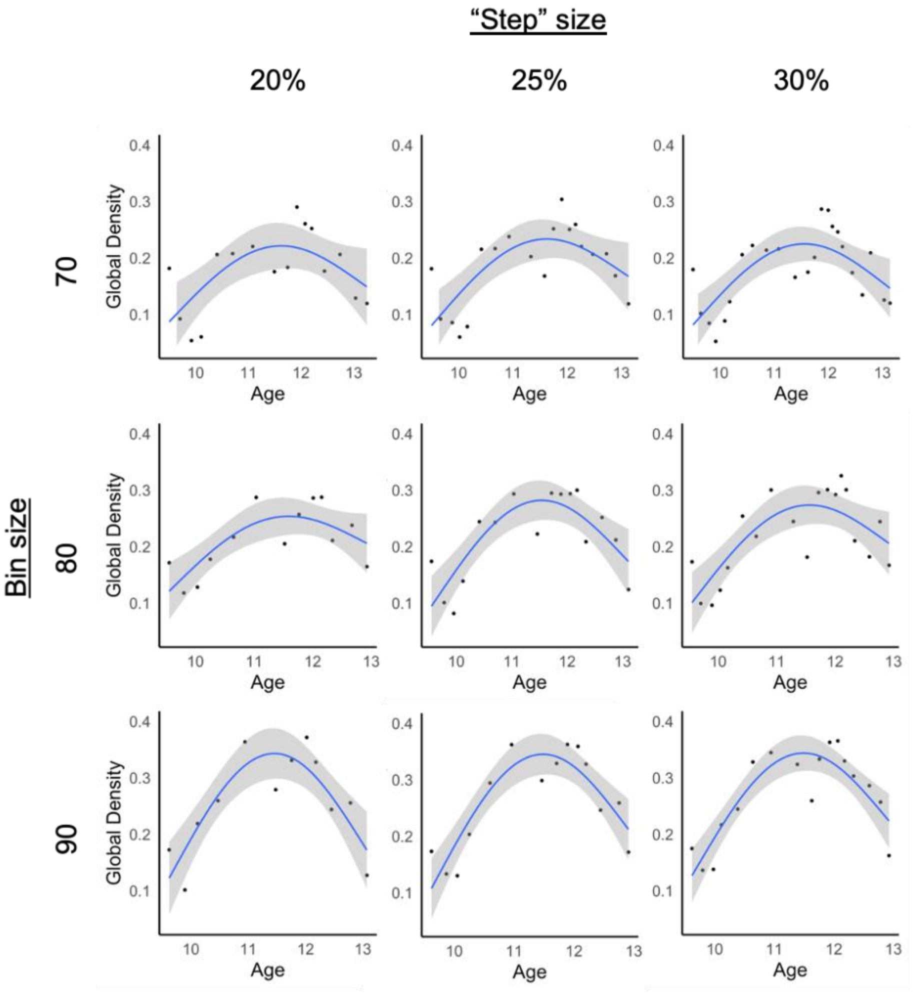

Figure S3. Age-related changes in global density for each sliding window configuration.

Table S2. Model comparisons and coefficients for nodal hubs

	Across configurations		Bin 80, Step 25%							
			AIC			s(Age)				
Regions	Best Fit	#	Null	Linear	Smooth	EDF	Ref DF	F	p	FDR
L_1	S	7	26.90	21.50	**13.30**	1.93	1.99	12.00	0.001	0.003
L_11l	N	5	28.00	24.60	**21.20**	1.84	1.97	6.64	0.015	0.029
L_2	N	9	**35.70**	36.60	36.60	1.00	1.00	1.03	0.326	0.389
L_31pd	S	9	48.60	50.40	**32.80**	1.97	2.00	15.40	0.000	0.001
L_31pv	S	5	31.20	33.20	**25.40**	1.92	1.99	5.04	0.023	0.043
L_3a	S	9	46.50	43.50	**28.90**	1.96	2.00	18.70	0.000	0.001
L_3b	L	9	36.10	**21.10**	21.20	1.08	1.16	23.60	0.000	0.001
L_4	L	9	43.60	**20.40**	20.40	1.00	1.00	53.60	0.000	0.000
L_45	L	7	30.10	**22.50**	22.50	1.00	1.00	11.60	0.004	0.010
L_47l	N	9	**30.80**	31.00	31.00	1.50	1.75	0.94	0.294	0.363
L_6d	S	5	25.00	25.50	**12.60**	1.96	2.00	11.00	0.001	0.004
L_7m	L	7	37.80	**27.70**	27.70	1.00	1.00	15.80	0.001	0.004
L_7PC	L	7	41.50	**29.70**	30.00	1.28	1.48	14.60	0.001	0.004
L_8Ad	L	6	39.20	**32.50**	32.50	1.00	1.00	10.10	0.006	0.014
L_8Av	N	8	**33.70**	33.90	33.90	1.00	1.00	1.65	0.220	0.307
L_8BM	L	7	51.20	**45.90**	45.90	1.49	1.74	4.30	0.027	0.050
L_8C	L	9	55.10	**42.90**	43.10	1.41	1.65	14.30	0.001	0.004
L_9.46d	N	8	**10.90**	11.60	11.60	1.00	1.00	1.24	0.284	0.356
L_9a	S	7	40.70	41.10	**23.10**	1.97	2.00	18.40	0.000	0.001
L_9m	N	9	**31.30**	31.70	31.70	1.00	1.00	1.47	0.245	0.318
L_9p	N	8	**35.40**	36.70	36.70	1.00	1.00	0.62	0.443	0.482
L_A1	L	9	42.40	**29.20**	29.40	1.14	1.26	18.70	0.000	0.003
L_a47r	L	9	30.40	**18.50**	18.50	1.00	1.00	19.50	0.000	0.003
L_A5	L	9	39.60	**26.00**	26.00	1.00	1.00	23.10	0.000	0.001
L_AIP	S	8	39.00	38.50	**29.80**	1.93	2.00	7.61	0.005	0.012
L_FOP4	N	8	**29.60**	30.00	30.00	1.00	1.00	1.50	0.241	0.318
L_FOP5	N	7	**32.50**	33.40	33.40	1.00	1.00	1.01	0.332	0.390
L_IFJa	N	7	**33.10**	33.60	33.60	1.00	1.00	1.37	0.261	0.333
L_LBelt	S	9	49.90	48.30	**39.80**	1.93	1.99	9.23	0.004	0.009
L_MBelt	S	6	37.50	21.80	**15.00**	1.91	1.99	28.80	0.000	0.000
L_p10p	L	7	39.90	**34.90**	32.90	1.77	1.95	5.35	0.014	0.028
L_p9.46v	N	8	**32.30**	29.50	29.70	1.42	1.66	4.04	0.081	0.133
L_PF	L	7	20.60	**11.00**	11.00	1.00	1.00	15.00	0.002	0.005
L_PFcm	S	9	36.10	33.60	**16.40**	1.97	2.00	22.10	0.000	0.000
L_PFt	S	9	47.30	43.80	**30.30**	1.96	2.00	16.80	0.000	0.001
L_PGi	S	5	**40.50**	42.10	38.40	1.85	1.98	2.47	0.109	0.172
L_TGd	S	6	34.70	33.40	**29.60**	1.85	1.98	4.14	0.031	0.057
L_TPOJ2	S	9	43.00	44.70	**32.80**	1.95	2.00	8.99	0.003	0.009
L_V2	N	8	**19.90**	21.00	21.00	1.00	1.00	0.81	0.382	0.435
L_V3	N	9	**26.60**	27.40	27.70	1.19	1.35	1.07	0.408	0.457
R_23c	S	7	51.50	29.70	**22.70**	1.91	1.99	42.50	0.000	0.000
R_31pv	N	7	**36.20**	36.40	36.70	1.35	1.58	1.68	0.308	0.374
R_3a	S	9	45.60	44.80	**37.00**	1.92	1.99	7.82	0.007	0.014
R_4	N	9	**16.10**	14.90	15.20	1.27	1.47	1.75	0.154	0.233
R_46	N	5	**11.90**	9.80	10.10	1.25	1.44	3.54	0.097	0.156
R_6a	N	6	**38.80**	37.10	37.10	1.00	1.00	3.63	0.077	0.132
R_6ma	S	9	45.80	36.00	**32.80**	1.83	1.97	11.00	0.001	0.004
R_6r	N	7	**35.50**	37.40	35.30	1.78	1.95	1.51	0.242	0.318
R_7m	S	5	43.20	**37.50**	35.60	1.77	1.95	5.79	0.011	0.023
R_8C	N	8	**27.60**	28.90	28.90	1.00	1.00	0.65	0.433	0.478
R_9.46d	N	9	**38.40**	40.30	40.30	1.00	1.00	0.01	0.932	0.936
R_9m	L	9	31.90	**16.90**	16.90	1.00	1.00	26.60	0.000	0.001
R_a10p	S	5	35.00	31.50	**26.20**	1.89	1.99	8.19	0.006	0.014
R_A5	L	5	36.00	**24.50**	23.00	1.74	1.93	11.10	0.001	0.004
R_AIP	N	7	**46.60**	46.70	46.70	1.00	1.00	1.69	0.214	0.307
R_IFSa	N	6	**33.20**	35.00	35.00	1.00	1.00	0.22	0.645	0.682
R_IP1	N	7	**37.40**	35.20	35.20	1.00	1.00	4.13	0.061	0.108
R_IP2	S	5	41.20	39.60	**25.30**	1.96	2.00	16.00	0.000	0.001
R_MBelt	N	6	**44.70**	44.00	44.00	1.00	1.00	2.62	0.127	0.196

R_OP4	S	9	54.50	55.40	**43.40**	1.95	2.00	9.61	0.002	0.006
R_p47r	L	5	40.40	**30.60**	30.60	1.01	1.03	14.80	0.002	0.005
R_PCV	S	8	47.20	40.80	**34.60**	1.90	1.99	12.30	0.001	0.004
R_PF	N	5	**31.70**	32.50	28.80	1.85	1.98	2.85	0.079	0.132
R_PFcm	N	5	**38.50**	40.50	40.50	1.00	1.00	0.01	0.936	0.936
R_PGi	N	5	**35.90**	35.40	35.50	1.12	1.22	1.72	0.178	0.263
R_PGp	N	7	**21.10**	21.80	22.00	1.08	1.15	0.87	0.340	0.393
R_RI	N	8	**25.40**	27.40	27.70	1.27	1.46	0.11	0.845	0.868
R_TE1a	N	8	**38.20**	39.80	39.80	1.00	1.00	0.33	0.573	0.615
R_TPOJ1	S	7	44.20	44.50	**33.40**	1.95	2.00	9.29	0.002	0.007
R_V1	N	6	**38.50**	39.60	38.00	1.74	1.93	1.40	0.227	0.311
R_V2	N	9	**27.90**	28.10	28.10	1.00	1.00	1.68	0.216	0.307
R_V3	N	9	**29.50**	31.40	31.40	1.00	1.00	0.08	0.780	0.813
R_V4	S	5	37.90	36.20	**28.10**	1.92	1.99	8.05	0.004	0.010
R_V6	L	9	38.80	**27.10**	27.10	1.00	1.00	19.00	0.001	0.003

NB: “Across configurations” refers to best-fitting models across the 9 window configurations. L: Linear, N: Null, S: Smooth, #: number of window configurations

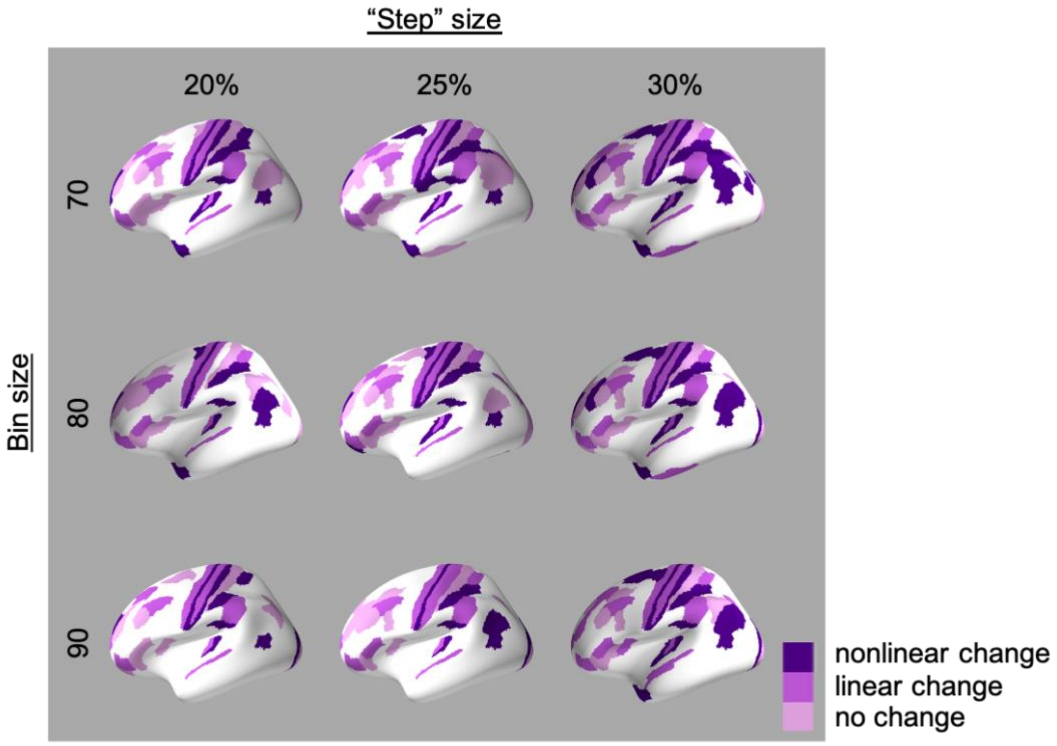

Figure S4. Age-related changes in standardized degree of hub regions for each sliding window configuration.

Table S3. Model comparisons and coefficients for modules

	Across configurations		Bin 80, Step 25%							
			AIC			s(Age)				
	Best Fit	#	Null	Linear	Smooth	EDF	Ref DF	F	p	FDR
Vis_Vis	L	9	1.83	**-8.95**	-8.95	1.00	1.00	17.11	0.001	0.004
SM_Vis	S	7	-6.58	-15.07	**-22.36**	1.92	1.99	14.74	0.000	0.002
SM_SM	L	5	14.90	**11.02**	9.86	1.71	1.91	5.53	0.033	0.046
DA_Vis	N	6	-31.79	**-35.52***	-35.45	1.48	1.72	4.84	0.053	0.080
DA_SM	N	9	**-15.97**	-14.11	-13.84	1.22	1.40	0.253	0.783	0.783
DA_DA	S	9	2.47	-0.54	**-15.85**	1.97	2.00	18.90	0.000	0.000
VA_Vis	S	7	-23.94	-22.00	**-29.61**	1.92	1.99	4.95	0.024	0.042
VA_SM	N	9	**-22.26**	-22.96	-23.04	1.53	1.78	1.43	0.191	0.223
VA_DA	L	9	-27.01	**-45.87**	-45.87	1.00	1.00	37.55	0.000	0.000
VA_VA	N	8	**-35.22**	-36.28	-36.28	1.00	1.00	2.95	0.108	0.126
Lim_Vis	N	9	**-22.43**	-20.84	-20.84	1.00	1.00	0.365	0.555	0.583
Lim_SM	L	9	-26.50	**-34.30**	-34.83	1.63	1.86	6.52	0.008	0.016
Lim_DA	N	6	**-43.07**	-44.41	-46.36	1.77	1.95	2.87	0.068	0.096
Lim_VA	N	9	**-32.93**	-31.61	-31.61	1.00	1.00	0.60	0.449	0.500
Lim_Lim	N	5	7.36	8.97	**1.79***	1.92	1.99	5.07	0.025	0.044
FP_Vis	S	8	-6.55	-4.56	**-16.77**	1.95	2.00	8.90	0.003	0.010
FP_SM	N	6	**-13.10**	-11.16	-13.97	1.82	1.97	1.86	0.184	0.223
FP_DA	S	5	-8.27	-21.89	**-26.70**	1.88	1.99	18.52	0.000	0.001
FP_VA	L	7	-24.57	**-35.46**	-35.17	1.26	1.45	13.41	0.002	0.008
FP_Lim	S	8	-20.85	-20.28	**-31.72**	1.95	2.00	9.82	0.002	0.009
FP_FP	S	7	-4.13	-4.95	**-15.84**	1.95	2.00	10.12	0.002	0.004
DM_Vis	S	6	-22.66	-24.53	**-34.28**	1.94	2.00	10.82	0.002	0.008
DM_SM	N	9	**-18.57**	-17.52	-20.18	1.81	1.96	2.08	0.137	0.179
DM_DA	L	8	-11.70	**-21.22**	-20.93	1.28	1.48	9.25	0.004	0.010
DM_VA	L	8	-41.12	**-46.54**	-46.54	1.00	1.00	8.26	0.012	0.030
DM_Lim	S	8	-21.59	-19.77	**-30.52**	1.95	2.00	7.79	0.006	0.014
DM_FP	S	5	-17.39	-15.64	**-22.68**	1.91	1.99	4.62	0.028	0.046
DM_DM	N	8	**-9.83**	-8.30	-9.93	1.75	1.94	1.70	0.259	0.259

NB: "Across configurations" refers to best-fitting models across the 9 window configurations. * Best-fitting model differed from most other sliding window configurations. DA: Dorsal Attention, DM: Default Model, FP: Frontoparietal, L: Linear, Lim: Limbic, N: Null, S: Smooth, VA: Ventral Attention, Vis: Visual, #: number of window configurations

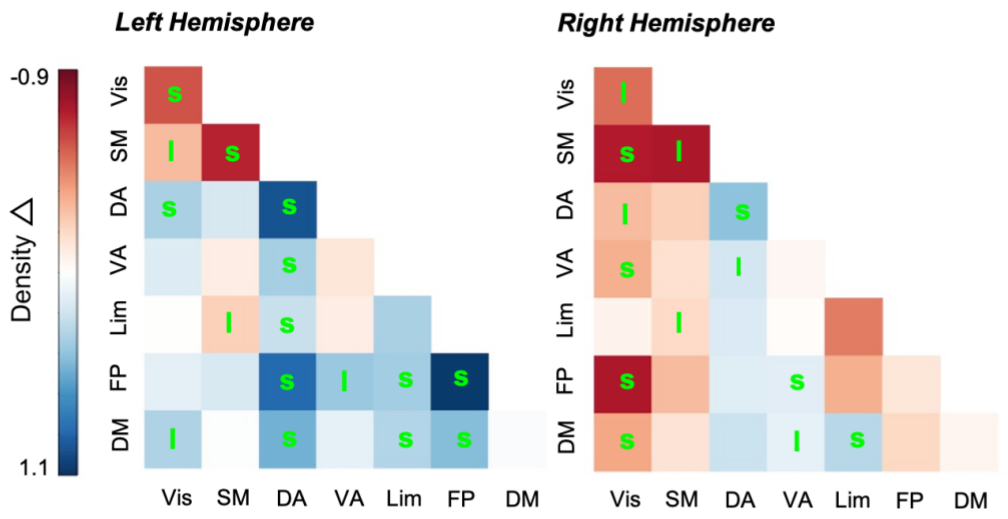

Figure S5. Changes in the density of connections for functional networks. *A)* Change in "normalized" density of connections between and within networks (*l* and *s* indicate significant linear and nonlinear trajectories, respectively). Effect size is the difference between maximum and minimum density for the sliding window size of 80 and step of 25%.

Table S4. Model comparisons and coefficients for modules examining with each sex

	Females										Males									
	Across configuration		Bin 80, Step 25%								Across configuration		Bin 80, Step 25%							
			AIC			s(Age)							AIC			s(Age)				
	Best Fit	#	Null	Linear	Smooth	EDF	Ref DF	F	p	FDR	Best Fit	#	Null	Linear	Smooth	EDF	Ref DF	F	p	FDR
Vis_Vis	N	8	**-5.6**	-5.01	-5.49	1.62	1.85	1.04	0.284	0.332	N	6	**17.69**	18.89	18.89	1.00	1.00	0.72	0.411	0.480
SM_Vis	S	9	-1.53	-8.48	**-41.51**	1.99	2.00	94.12	0.000	0.000	N	9	**-1.75**	-2.93	-2.93	1.00	1.00	3.07	0.101	0.133
SM_SM	S	7	23.76	25.24	**10.01**	1.97	2.00	12.95	0.001	0.002	N	8	**25.34**	22.99	23.26	1.34	1.56	2.40	0.095	0.133
DA_Vis	N	9	**-18.65**	-16.76	-16.76	1.00	1.00	0.09	0.767	0.805	N	6	-3.39	**-7.83***	-7.56	1.21	1.38	5.82	0.032	0.067
DA_SM	N	9	**-2.85**	-0.92	-3.24	1.79	1.96	1.58	0.226	0.297	N	7	-26.86	-25.06	**-33.48**	1.93	1.99	5.64	0.016	0.059
DA_DA	S	8	11.49	6.46	**-3.02**	1.94	2.00	13.20	0.000	0.002	S	8	0.77	2.59	**-17.8**	1.98	2.00	19.67	0.000	0.000
VA_Vis	N	9	**-25.21**	-24	-23.72	1.23	1.40	0.37	0.526	0.581	N	9	**-22.47**	-21	-21.77	1.66	1.89	0.86	0.364	0.403
VA_SM	N	9	**-12.79**	-12.75	-12.75	1.00	1.00	1.83	0.198	0.277	N	9	**-8.56**	-7.87	-11.46	1.84	1.98	2.83	0.079	0.127
VA_DA	L	7	-5.99	**-32.76**	-33.7	1.68	1.90	40.06	0.000	0.000	N	9	**-41.91**	-40	-44.53	1.87	1.98	3.01	0.088	0.129
VA_VA	N	8	**-8.62**	-7.87	-8.71	1.67	1.89	1.16	0.262	0.332	S	8	1.09	-0.22	**-6.33**	1.90	1.99	5.97	0.011	0.020
Lim_Vis	N	9	**-13.33**	-11.87	-16.03	1.86	1.98	2.81	0.085	0.149	N	8	**-27.35**	-25.35	-25.3	1.48	1.73	0.34	0.682	0.682
Lim_SM	S	5	-16.12	-18.25	**-22.49**	1.86	1.98	5.05	0.018	0.038	N	5	-5.21	**-9.38***	-9.38	1.00	1.00	6.60	0.022	0.059
Lim_DA	N	9	**-28.76**	-28.27	-28.27	1.00	1.00	1.38	0.259	0.320	N	9	**-30.34**	-32.69	-32.66	1.02	1.03	4.18	0.057	0.109
Lim_VA	N	6	**-27.18**	-27.23	-27.23	1.00	1.00	1.91	0.189	0.277	N	7	-17.57	**-20.77***	-20.51	1.35	1.57	4.41	0.065	0.114
Lim_Lim	N	6	24.84	**21.78***	22.05	1.23	1.41	4.43	0.061	0.143	S	5	4.14	0.16	**-10.9**	1.95	2.00	15.04	0.000	0.001
FP_Vis	S	8	4.3	5.89	**-9.5**	1.97	2.00	12.76	0.001	0.003	N	9	**-16.36**	-14.53	-18.41	1.85	1.98	2.70	0.112	0.138
FP_SM	S	5	-22.11	-20.14	**-27.58**	1.92	1.99	4.89	0.026	0.050	N	9	**12.92**	14.85	13.28	1.74	1.93	1.34	0.312	0.364
FP_DA	S	5	-12.34	-20.52	**-24.87**	1.87	1.98	10.58	0.001	0.004	L	5	-5.38	-12.32	**-16.84***	1.87	1.98	9.46	0.002	0.020
FP_VA	L	9	-18.59	**-34.98**	-34.98	1.00	1.00	30.19	0.000	0.000	N	7	-12.21	**-17.77***	-17.49	1.32	1.53	6.68	0.022	0.059
FP_Lim	L	6	-4.39	**-9.4**	-9.4	1.00	1.00	7.69	0.015	0.034	N	7	**-6.54**	-5.71	-9.07	1.84	1.97	2.61	0.092	0.129
FP_FP	N	9	**-10.79**	-8.79	-11.23	1.80	1.96	1.69	0.222	0.332	L	9	9.84	**-4.45**	-4.45	1.00	1.00	24.76	0.000	0.001
DM_Vis	N	9	**-20.96**	-19	-19	1.00	1.00	0.03	0.859	0.859	N	8	-21.63	-23.23	**-27.97***	1.87	1.98	6.04	0.018	0.059
DM_SM	S	5	-29.43	**-34.27***	-37.22	1.82	1.97	6.03	0.010	0.026	N	9	**-0.51**	1.46	1.14	1.59	1.83	0.53	0.559	0.587
DM_DA	S	6	-33.1	-39.62	**-44.32**	1.87	1.98	9.22	0.002	0.006	L	7	-6.94	**-12.96**	-13.04	1.53	1.78	4.87	0.019	0.059
DM_VA	L	9	-25.35	**-38.81**	-40.96	1.78	1.95	14.29	0.000	0.001	N	8	-29.98	-31.45	**-42.62***	1.95	2.00	11.91	0.001	0.020
DM_Lim	N	9	**-24.07**	-22.89	-22.89	1.00	1.00	0.74	0.405	0.473	N	6	0.03	-0.32	**-8.08***	1.92	1.99	7.32	0.008	0.059
DM_FP	N	5	**-25.73**	-25.92	-27.18	1.72	1.92	2.75	0.148	0.239	N	7	**-4.15**	-6.71	-9.4	1.81	1.96	4.15	0.030	0.067
DM_DM	N	9	**-11.41**	-9.98	-9.9	1.47	1.72	0.88	0.536	0.536	N	9	**5.94**	7.65	7.78	1.44	1.69	0.62	0.625	0.625

NB: "Across configurations" refers to best-fitting models across the 9 window configurations. * Best-fitting model differed from most other sliding window configurations. DA: Dorsal Attention, DM: Default Model, FP: Frontoparietal, L: Linear, Lim: Limbic, N: Null, S: Smooth, VA: Ventral Attention, Vis: Visual, #: number of window configurations
